# Insight into the Structure–Odor Relationship of Molecules: A Computational Study Based on Deep Learning

**DOI:** 10.3390/foods11142033

**Published:** 2022-07-09

**Authors:** Weichen Bo, Yuandong Yu, Ran He, Dongya Qin, Xin Zheng, Yue Wang, Botian Ding, Guizhao Liang

**Affiliations:** Key Laboratory of Biorheological Science and Technology, Ministry of Education, Bioengineering College, Chongqing University, Chongqing 400044, China; 201819021012@cqu.edu.cn (W.B.); 202019021097t@cqu.edu.cn (Y.Y.); 202119131220@cqu.edu.cn (R.H.); 20161901020@cqu.edu.cn (D.Q.); 201819021020@cqu.edu.cn (X.Z.); 201919021037@cqu.edu.cn (Y.W.); 201919021030@cqu.edu.cn (B.D.)

**Keywords:** odor prediction, hazardous molecules, multilayer perceptron (MLP), convolutional neural network (CNN)

## Abstract

Molecules with pleasant odors, unacceptable odors, and even serious toxicity are closely related to human social life. It is impractical to identify the odors of molecules in large quantities (particularly hazardous odors) using experimental methods. Computer-aided methods have currently attracted increasing attention for the prediction of molecular odors. Here, through models based on multilayer perceptron (MLP) and physicochemical descriptors (MLP-Des), MLP and molecular fingerprint, and convolutional neural network (CNN), we conduct the two-class prediction of odor/no odor, fruity/no odor, floral/no odor, and woody/no odor, and the multi-class prediction of fruity/flowery/woody/no odor on our newly refined molecular odor datasets. We show that three kinds of predictors can robustly predict molecular odors. The MLP-Des model not only exhibits the best prediction results (the AUC values are 0.99 and 0.86 for the two- and multi-classification models, respectively) but can also well reflect the characteristics of the structure–odor relationship of molecules. The CNN model takes 2D molecular images as input and can automatically extract the structural features related to molecular odors. The proposed models are of great help for the prediction of molecular odorants, understanding the underlying relationship between chemical structure and odor perception, and the discovery of new odorous and/or hazardous molecules.

## 1. Introduction

In daily life, all kinds of molecular odors, including the attractive aromas of food and spices, the special odors, such as deodorant, body spray, soap, lotion and laundry liquid, or the rotten smell or unpleasant smells in garbage, not only affect our emotions, memories and behaviors but also have a certain impact on biological evolution [1]. The identification of various odors, particularly toxic odors, can guide people to avoid harm and can expand the application of molecules with specific odors and help to develop new materials, food, cosmetics, and so on.

In the past, researchers generally identified the odor of compounds through sensory experiments; however, large-scale sensory evaluation tests require a great deal of time and effort. In recent years, researchers have used electronic noses to test molecular odors and even designed manual feature extractors to predict the odor characteristics of molecules. Otherwise, the above method depends on manual design and designer’s experience, with the poor universality and missing prediction in real time [2]. At present, computational methods have attracted increasing attention in detecting the structure–odor relationship of molecules. 

It is worth mentioning that the structure–odor relationship model can establish a link between molecular structure characteristics and odor perceptions. It can also be employed to identify toxic odors emitted by hazardous materials and the discovery odorous molecules [3]. Due to the rapid development of high-performance computing and molecular datasets, deep-learning techniques have been successfully used for the prediction of molecular odors. Zhang et al. [4] combined a deep belief network (DBN), convolutional neural network (CNN) and recurrent neural network (RNN) to predict molecular odors and developed a mixed integer linear/nonlinear programming model with an average accuracy of 92% to detect the physicochemical properties of odorous molecules. 

Wu et al. constructed a CNN-based model for odor prediction using the response signal generated by an electronic nose, achieving a 99.9% accuracy in distinguishing between pleasant and unpleasant odors. Zhang et al. [5] developed an artificial intelligence platform based on random forest (RF) and DBN methods to predict the color and odor of molecules, and the prediction accuracy was up to 94.75% ± 0.44%. Sharma et al. [6] proposed a DBN-based DeepOlf model to predict both potential odorants and their interactions with related receptors. 

Another study by Sharma et al. [7] focused on the multi-label prediction of odorous molecules by using the models based on physicochemical descriptors and DBN, molecular fingerprints and DBN, and molecular images and CNN. The proposed models based on molecular fingerprint and DBN, and molecular image and CNN presented an accuracy of 97.3% and 98.3% on the independent test set, respectively. In Tran et al. [8], the molecules were fed into the network as a 3D object to enable the training of the encoder, and they finally obtained the DeepNose classifier for odor prediction. 

A set of descriptors for the target chemicals was successfully predicted by a series of computer simulations by Nozaki et al. [9]. The models with prediction accuracies of 53% for true positives and of 85% for true negatives were constructed based on the descriptors. In Benjamin Sanchez-Lengeling et al. [10], a graphical neural network was trained to predict the relationship between the molecular structure and its odor, and the performance of the model was validated on multiple public datasets. In the study of Kowalewski and Ray et al. [11], the modeling of chemical features combined with odor receptor activity data enabled odor prediction.

To date, deep learning has achieved some pioneering results; however, it is still in the early exploration stage in the field of odor prediction; moreover, few studies have focused on the structure–odor relationship modeling of molecules. Deep-learning modeling requires high-quality molecular odor datasets as well as appropriate structural features that are closely related to molecular odors to ensure the predictive ability of the model. Certainly, it is necessary to improve the prediction accuracy of molecular odor; meanwhile, the in-depth investigation on the structure–odor relationships of molecules should be emphasized.

**Figure 1:**
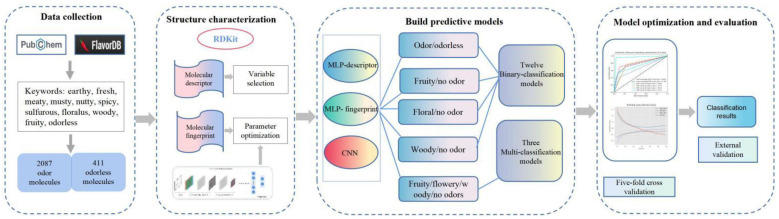
The flowchart of the modeling process. First, dataset was collected from FlavorDB and PubChem databases. Second, RDKit was used to generate the molecular descriptors, fingerprints and 2D images. Third, based on three nonlinear models (MLP-descriptor, MLP-fingerprint, and CNN), 15 predictive models were built for five classification tasks. Finally, a five-fold cross-validation was used to optimize the modeling parameters, and an external validation was used to evaluate the model.

Here, according to Figure 1, we propose three novel kinds of models based on multilayer perceptron (MLP) and physicochemical descriptor (MLP-Des), MLP and fingerprint (MLP-Fin), and CNN to conduct the two-class prediction for odor/no odor, fruity/no odor, floral/no odor, woody/no odor, and the multi-class prediction for fruity/floral/woody/no odor. We further explored the structure–odor relationship of molecules based on the MLP-Des model, thereby, obtaining molecular physicochemical descriptors that can facilitate the identification of odor characteristics. The deep-learning-based structure–odor relationship model can help in the prediction of molecular odors, understanding the underlying mechanisms, and in the identification of hazardous molecular odors and novel molecules with specific odors in the environment.

## 2. Methods

### 2.1. Dataset Collection and Preparation

The molecular odor datasets were retrieved from FlavorDB [12] and PubChem databases [13]. The used keywords were “eary (126)”, “fresh (160)”, “meaty (103) “, “musty (96)”, “nutty (142)”, “spicy (158)”, “sulfurous (115)”, “woody (240)”, and “fruity (640)” in searching odorous molecules and “odorless” in searching odorless molecules. All the molecules collected were corrected according to the recorded structures in the PubChem database to ensure accuracy. As shown in Table 1, the dataset contained 2087 odorous molecules and 411 odorless molecules.

First, the positive and negative samples were assembled and then randomly disordered. Then, all the samples were divided into training set and test set according to 4:1, where the training set was used for modeling and the test set was employed to evaluate the predictive ability of the model. Additionally, the training set were divided into five sets for 5-fold cross-validation [14] or hold-out validation [14], and evaluation processes were repeated 10 times to ensure the accuracy of the modeling results. For the two-classification, a 5-fold cross-validation method was applied to optimize the parameters to obtain the model. For the multi-classification, a hold-out validation was used to evaluate the results of each training to acquire the optimal modeling parameters

### 2.2. Molecular Structure Characterization

**Figure 2:**
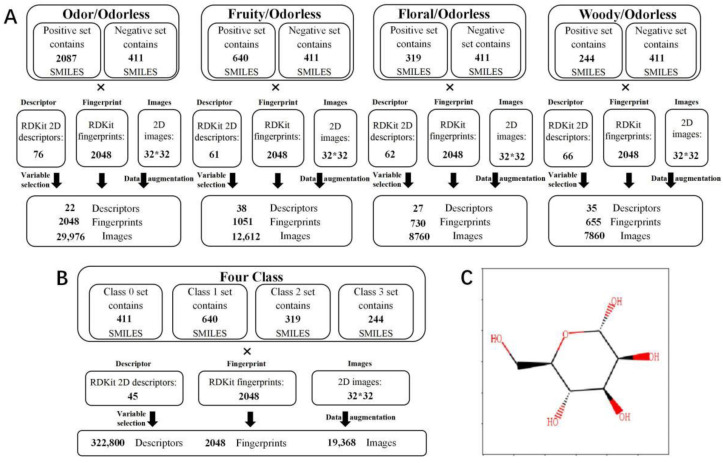
Structural characterization for the molecular odor dataset. (**A**) The structural parameters (fingerprints, descriptors, and 2D images) of dataset used for two-class (odor/odorless, fruity/odorless, floral/odorless, and woody/odorless) prediction. (**B**) The structural parameters (fingerprints, descriptors, and 2D images) of dataset used for four-class (fruity/flowery/woody/odorless) prediction, where class 0 set, class 1 set, class 2 set, and class 3 set present odorless, fruity, floral, and woody molecules, respectively. (**C**) The example image in 2D images used for CNN modeling.

As shown in Figure 2, molecular structures were characterized by molecular descriptors, molecular fingerprints, and molecular images. The open-source chemical information package, RDKit [15], was applied to generate the molecular descriptors (Table S1), fingerprints and 2D images. The SMILES string of molecules was utilized as input to characterize the 2D image while distinguishing the stereoisomeric features of molecules.

### 2.3. Feature Selection

For the MLP-Des model, variable loading analysis was applied for feature screening. First, the features with repeated meanings were removed according to the eigenvalues of the correlation coefficient matrix, and then the rotated component matrix was obtained by oblique rotation [16]. The 75% variance of the original variable matrix was used as the common factor selection standard, and then the features with large contribution to the factor were obtained through the factor load distribution. For the length feature screening of molecular fingerprint, from 256 to 2048, the difference increased by 100, and the length with the best prediction result was selected to build the model. Feature selection used IBM SPSS statistics software [17].

### 2.4. Multilayer Perceptron

Generally, MLP (which consists of an input layer, multiple hidden layers, and an output layer) updates the parameters by a back propagation algorithm and optimizes the model by a gradient descent algorithm. Here, the binary-classification MLP models were composed of three fully connected layers, and the weights were initialized by uniform distribution. The output layer used a Sigmoid activation function, while the rest used a ReLU activation function. In parallel, the multi-classification MLP models encompassed an input layer, two fully connected layers, and a four classified Softmax layer output layer [18]. The two fully connected layers of the MLP-Des model contained 128 and 64 hidden neurons, respectively, and the two fully connected layers of the MLP-Fin model included 32 hidden neurons.

### 2.5. Two-Dimensional Convolutional Neural Network

**Figure 3:**
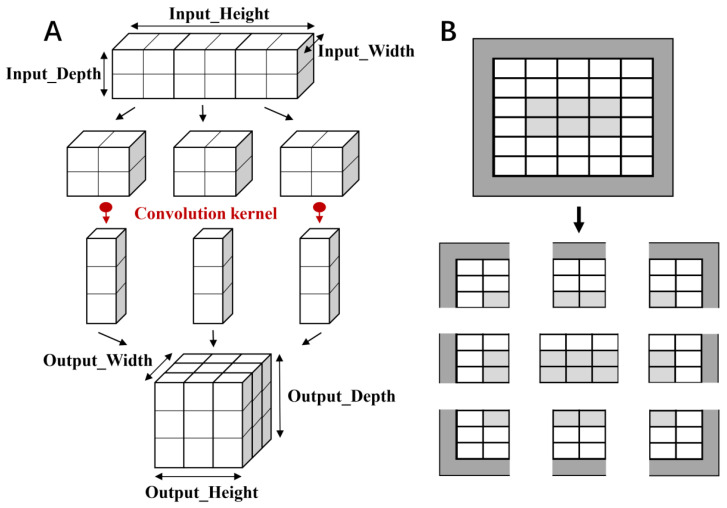
Schematic diagram of CNN. (**A**): In the convolution layer, the convolution operation was used to extract the feature of input data derived from the Depth, Height, and Width. The dimensions of the output data were controlled by changing the size of convolutional kernel and stride of the convolution operation. (**B**): To speed up the calculation, prevent over-fitting, and improve the generalization of the model, the pooling operation was used to compress data dimensions and maintain the key information of data.

The basic component of CNN included a convolution layer, a pooling layer, and a full connected layer (Figure 3). The convolution layer extracted feature blocks from the input feature map and converted them into the output feature map. The pooling layer reduced the size of spatial information, and the features were mapped onto the samples using the fully connected layer. In the CNN-based binary-classification model, the convolution kernel size of three-layer convolution layer was set to (3, 3), and two maximum pooling layers were used for down-sampling with the size of 14 × 14. 

The ReLU activation function and the Sigmoid activation function were used for the intermediate layer and the output layer, respectively. The CNN-based multi-classification model was composed of five convolution layers and three pooling layers, and the last part was composed of two fully connected layers. The five convolution layers contained 32, 64, 64, 128, and 128 hidden neurons, respectively, and the fully connected layer contained 512 hidden neurons. The ReLU activation function was used for the intermediate layer, while the Softmax activation function was applied for the final fully connected layer, with the output value being the probabilities of the four categories.

### 2.6. Model Evaluation

A series of statistical parameters, including the Accuracy, Precision, Sensitivity, Specificity, Area under curve (*AUC*) by receiver operating characteristics (*ROC*) [19], and Matthews correlation coefficient (*MCC*) [20], were used to evaluate the predictive ability of the proposed model. The formulas for each parameter were as follows:

(1) $$\mathrm{Accuracy}=\frac{\mathrm{TP}+\mathrm{TN}}{\mathrm{TP}+\mathrm{FP}+\mathrm{TN}+\mathrm{FN}}$$

(2) $$\mathrm{Precision}=\frac{\mathrm{TP}}{\mathrm{TP}+\mathrm{FP}}$$

(3) $$\mathrm{Sensitivity}=\frac{\mathrm{TP}}{\mathrm{TP}+\mathrm{FN}}$$

(4) $$\mathrm{Specificity}=\frac{\mathrm{TN}}{\mathrm{TN}+\mathrm{FP}}$$

(5) $$\mathrm{MCC}=\frac{\mathrm{TP}\times \mathrm{TN}-\mathrm{TP}\times \mathrm{FN}}{\sqrt{\left(\mathrm{TP}+\mathrm{FP}\right)\times \left(\mathrm{TP}+\mathrm{FN}\right)\times \left(\mathrm{TN}+\mathrm{FP}\right)\times \left(\mathrm{TN}+\mathrm{FN}\right)}}$$

where TP (true positive) is the number of positives for correct classification; TN (true negative) is the number of negatives for correct classification; FP (false positive) is the number of negatives for incorrect classification; and FN (false negative) is the number of positives for incorrect classification.

## 3. Results

### 3.1. The MLP-Des Model Exhibited the Highest Predictive Capability

**Figure 4:**
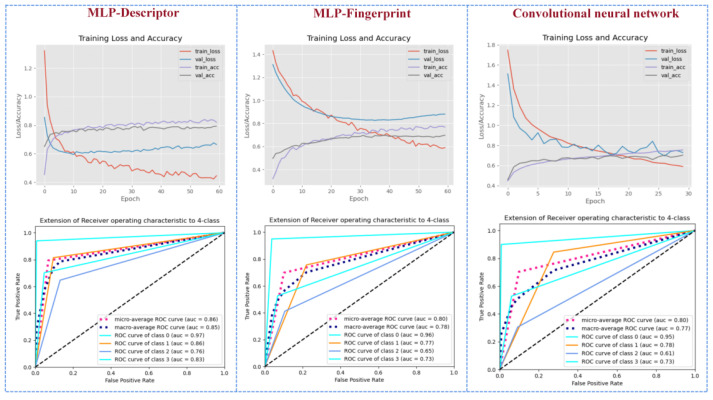
The multi-class prediction results of molecular odors. The “ROC curve of class 0” indicates the predicted value of odorless molecules; “ROC curve of class 1” denotes the predicted value of fruity molecules; “ROC curve of class 2” signifies the predicted value of floral molecules; “ROC curve of class 3” represents the predicted value of woody molecules; “micro-average ROC curve” suggests the micro-average predicted value of the model; and “macro-average ROC curve” shows the macro-average predicted value of the model.

Based on the parameters (Table S2) optimized by grid search and the Earning stopping method, we constructed 15 models of MLP-Des, MLP-Fin, CNN for the prediction of odor/no odor, fruity/no odor, floral/no odor, and woody/no odor, as well as no/fruity/flowery/woody odor, respectively. As shown in Table 2, the MLP-Des model showed better prediction performances compared with the MLP-Fin model and the CNN model. For the prediction of odor/no odor, fruity/no odor, and woody/no odor, the MLP-Des model generated the highest AUC value of 0.99 on the test set (Figure 4), while for the prediction of no/fruity/floral/woody odor, the MLP-Des model exhibited the highest accuracy of 0.800 on the test set (Table 3).

Through comparison, the MLP-Fin model had the highest predictive ability at a length of 2048 for RDKFP [15], with an *accuracy* of 0.98 (Table 2) and an *AUC* of 0.99 (Figure 4) for the prediction of odor/no odor on the independent test set. The multi-classification based on the MLP-Fin model generated the highest ability to identify odorless molecules (*AUC* of 0.96) (Figure 4).

As displayed in Table 2, the CNN-based model generated *accuracy* values larger than 94.0% and *AUC* values greater than 0.98 for the prediction of odor/no odor, fruity/no odor, floral/no odor, and woody/no odor in the test set, suggesting that the model can better solve the issue of imbalance between odorous and odorless samples (Figure 4). Moreover, the *AUC* value by the CNN model was larger than 0.80 (Table 3) for the prediction of the no/fruity/flowery/woody odor on the test set. The above results indicated that CNN only took the 2D molecular image as the input, omitting the steps of manual extraction and screening of characteristic variables, thereby, achieving the accurate prediction of molecular odors.

### 3.2. The MLP-Des Model Showed Better or Equivalent Predictions Compared with Exiting Models

We compared the prediction results of molecular odors between the models established in this study and the methods reported in the literature. As shown in Table 4, the prediction *accuracy* of the MLP-Des model was higher than that of AI-RF/DBN (*Accuracy* = 0.94) [5], GA-ANN (*Accuracy* = 0.90) [21], MILP/MINLP (*Accuracy* = 0.75) [4] and Olfactometer (*Accuracy* = 0.97) [22].

It was worth mentioning that the SOR model listed in Table 4 [7] applied DBN and CNN methods to predict the multi-label odors of molecules, whereas we achieved binary-classification and single-label multi-class prediction of molecular odors. The compared results exhibited that the *AUC* value of the multi-class prediction based on the MLP-Des model was equivalent to that of the SOR model.

### 3.3. The Structure–Odor Relationship Derived from the MLP-Des Model

Our MLP-Des model had the highest prediction ability, which was equivalent to or better than the results of the models listed in Table 4; moreover, this model can quantitatively describe the structure–odor relationship of molecules.

**Figure 5:**
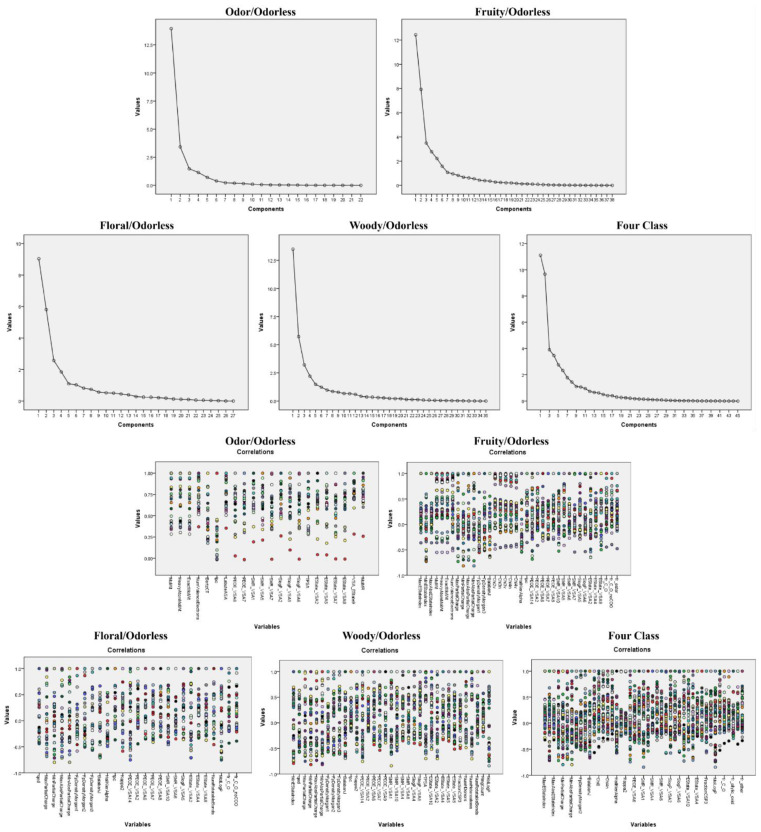
The five figures on top represent the main factors extracted by variable loading analysis of the molecular descriptors. The components with high values shown in the figures are the common factors. The five graphs below are the correlation coefficient matrixes. This shows all variables for each model. Variables with high correlation formed a component. Four, seven, six, six, and ten common factors, which explain more than 75% variance of the original variable matrix, were extracted for prediction of two−class (odor/odorless, fruity/odorless, floral/odorless, and woody/odorless) and four−class (odorless/fruity/floral/woody) molecules, respectively. The main factors are the most important set of molecular descriptor features that are beneficial to the identification of a certain odor.

***Molecular weight, electronegativity, and surface interactions were of great importance for odor recognition.*** In the odor/odorless prediction model, four extracted common factors explained more than 75% variance (Figure 5). Except Ipc (branch in molecule), the other descriptors significantly contributed to the first common factor (the contribution rate was larger than 0.5, Table S3). Hence, we focused on the physicochemical significance of variables that contributed to the first common factor. 

Among them, molar refractive index (MolMR), atomic number (HeavyAtomCount), and molecular weight (HeavyAtomMolWt, ExactMolWt, and MolWt) were molecular composition descriptors; VSA_EState9, PEOE_VSA, and EState_VSA were related to the electronegativity and electrostatic interaction of the backbone atoms; LabuteASA, SlogP_VSA, SMR_VSA, and TPSA described the surface area, hydrophobic interaction, hydrophilic interaction, and polarizability. BertzCT was a quantitative indicator of the molecular complexity. Therefore, the molecular weight, electronegativity, and surface interactions played crucial roles in characterizing odorous molecules.

***Molecular charge, surface interaction, and shape were practicable for the identification of fruity odors.*** Seven common factors conducive to identifying fruity molecules were obtained by variable load analysis (Figure 5). We analyzed the variables (Table S4) with large significant loading (mainly greater than 0.5) on the first two common factors. 

A total of 15 descriptors contributed significantly to the common factor 1, while a total of 11 descriptors had great impact on the common factor 2. The variables that contributed greatly to the common factor 1 were partial charges (NumValenceElectrons), molecular weights (ExactMolWt, MolWt, and HeavyAtomMolWt), atomic and valence electronic information (Chi-like descriptors), SMR_VSA (polarizability), PEOE_VSA (direct electrostatic interactions), and EState_VSA (electronegativity of skeleton atoms). 

The common factor 2 mainly reflected the characteristics of eight variables involving local charge (MaxPartialCharge, MaxAbsPartialCharge, MinAbsPartialCharge, and MinPartialCharge), charge information (MinEStateIndex), the molecular shape used to characterize the graphic descriptors (BalabanJ), HallKierAlpha, and Morgan fingerprint density (FpDensityMorgan 3). Thus, the features, such as molecular charge, surface interaction, and shape can be applied to identify fruity molecules.

***Molecular hydrophobic characteristics, composition, and charge were helpful in the identification of floral odors.*** A total of six common factors were extracted from the molecular descriptors in the floral/no odor prediction model (Figure 5). Ten descriptors (Table S5) had a significant impact on the loading of the common factor 1, including direct electrostatic interactions (PEOE_VSA), charge information (EState_VSA), local charge (MaxPartialCharge, MaxAbsPartialCharge, MinAbsPartialCharge, and MinPartialCharge), polarizability (SMR_VSA), lipid–water partition coefficient (MolLogP), graphical descriptors (BalabanJ) and metrics of drug similarity (Qed). 

Seven descriptors (Table S5) contributed significantly to the common factor 2, including the descriptors for fragment counting (fr_C_O and fr_C_O_noCOO), branching results in the molecule (Ipc), the number of rotatable bonds (NumRotatableBonds), kappa 2 characterizing the molecular shape, hydrophobic and hydrophilic interactions (SlogP_VSA5), and molecular shape (HallKierAlpha). In summary, molecular hydrophobic characteristics, composition, and charge properties played a vital role in the identification of floral odors.

***Molecular surface interactions, charge and shape promoted the identification of woody odors.*** Through variable load analysis, six common factors were obtained to identify woody/odorless odors (Figure 5). What we primarily analyzed was the characteristics of variables with large loading on the common factor 1 and the common factor 2 (Table S6). The common factor 1 was mainly characterized by the following variables: surface interactions, charge-related descriptors, graphical descriptors (BalabanJ), indicators of drug similarity (QED) and Morgan fingerprint density (FpDensityMorgan1). The common factor 2 refined the physicochemical parameters, including the number of rotatable bonds (Num Rotatable Bonds), the molecular shape (kappa2), the branching result in the molecule (Ipc), the number of rings (RingCount) and the proportion of sp^3^ hybridized carbon atoms (FractionCSP3). Therefore, molecular surface interactions, charge and molecular shape boosted the identification of woody odors.

***Molecular charge, composition, shape, and surface interaction were suitable for multi-classification of molecular odors.*** In the multi-classification model, total ten common factors were extracted (Figure 5). The results of variable loading analysis (Table S7) exhibited that the variables significantly contributing to the common factor 1 encompassed lipid–water partition coefficient (MolLogP), polarizability (SMR_VSA), hydrophobic and hydrophilic interaction (SlogP_VSA), molecular polar surface area (TPSA), charge information (PartialCharge, PEOE_VSA, MaxAbsEStateIndex MinEStateIndex and ESTate_VSA), Morgan fingerprints (FpDensityMorgan), and graphical descriptors (BalabanJ). 

The variables that had great influence on the common factor 2 mainly included the molar refractive index (MolMR), atomic and valence electronic information (Chi-like descriptors), molecular complexity (BertzCT), molecular fragment counts (fr_ether, fr_C_O, fr_ester, fr_C_O_noCOO, and fr_allylic_oxid), rotatable bond count (NumRotatableBonds), proportion of sp^3^ hybridized carbon atoms (FractionCSP3), characterized molecular shape (HallKierAlpha and kappa), and Morgan fingerprint (FpDensityMorgan) descriptors. In conclusion, the variable characteristics, including the molecular charge, composition, shape, and surface interaction, were beneficial to the multi-classification of molecular odors.

## 4. Discussion

The prediction of molecular odor based on computational methods has important practical significance for discovery of molecules with specific odors, in particular molecules with hazardous odors. The prediction modeling of molecular odor first requires high-quality molecular odor datasets. In recent years, the public molecular odor database has become an important source of datasets as used in this study. We combined the multi-step screening strategy to establish the molecular odor dataset (Table 1), which was utilized to establish the two-classification and multi-classification models of molecular odor based on deep-learning methods.

Our models based on deep-learning algorithms can well predict the odorous/odorless, fruity/odorless, floral/odorless, and woody/odorless characteristics of molecules. The models can be used to detect the odor of molecules and identify the molecules with specific odors to meet the need of food flavor, cosmetic fragrance, material preparation, etc. More importantly, the proposed models can be used to discern the molecules with toxic odor, and to clarify the structure–odor relationship of molecules, thereby, understanding the underlying mechanism targeting different odor receptors.

Over-fitting is an issue to be solved in deep learning. Here, we took multiple steps to avoid overfitting. First, the generalization ability of the model was evaluated by a 5-fold cross validation, and the predictive ability of the model on the test set was evaluated by *MCC*, *AUC*, and other indicators. Second, over-fitting was further avoided by the following means: (i) Molecular images were widely collected from multiple databases, and the datasets were cut, flipped, and illuminated to increase the diversity of samples. (ii) The weight was processed by L2 regularization to punish the features that were not important for molecular odors. (iii) Dropout was used to reset weight of some neurons in each training process to reduce the amount of parameters and avoid over-fitting. (iv) The training was terminated in advance by early stopping to avoid the increase of error rate on test set on the account of the excessive learning of the characteristics of training set, thereby, obtaining the best generalization model.

The MLP-Des model not only had the highest prediction ability for molecular odors but could also quantitatively describe the molecular structure–odor relationship. The structure–odor relationship investigations revealed that charge information played a crucial role in the identification of molecular odors in both the binary-classification (odor/no odor, fruity/no odor, floral/no odor, and woody/no odor) and the multi-classification (no/fruity/flowery/woody odor). Moreover, it is noteworthy that the characteristics representing four kinds of molecular odors cover those of the binary-classification prediction of molecular odors—that is, the characteristics conducive to identifying a single odor are also beneficial to identifying multi-class odors, which demonstrates the reliability of the extracted odor recognition characteristics from the side.

The CNN model based on 2D molecular images exhibited high accuracy and stability for predicting molecular odors. In this study, the SMILES string was used to generate molecular image, which is mainly composed of two parts: the molecular pixel and background color. Molecular pixel is the key feature that the model needed to learn (about 10% of the whole image area), while the background color is invalid input or noise interference (about 90%). When image resolution is too low, the image is too fuzzy to effectively reflect the characteristics of molecular pixel. With the continuous improvement of resolution, the molecular pixel features are easier to be learned by the model. However, it must be noted that the increased area of molecular pixel is much smaller than that of background color every time resolution is improved. If the resolution is increased and transited, the dimension of input variables will soar up, resulting in many invalid background interference inputs, which will raise the difficulty of model training. 

Thus, the selection of molecular image resolution must be a process of gradual increase and then gradual decrease, and there is a peak optimal value. We attempted to construct images with different resolutions from 30 × 30 to 300 × 300; as a result, the prediction results are the best when the resolution is 32 × 32. The model can not only precisely identify the key molecular features related to odors but also efficiently deal with the interference of background color noise. Furthermore, CNN has the advantage in image recognition, that is, it can extract the key features of molecules without special high resolution to achieve high predictions. Therefore, CNN can automatically extract structural information related to molecular odors, thereby, avoiding the manual input of molecular structural parameters and obtaining a robust prediction model for molecular odors.

**Figure 6:**
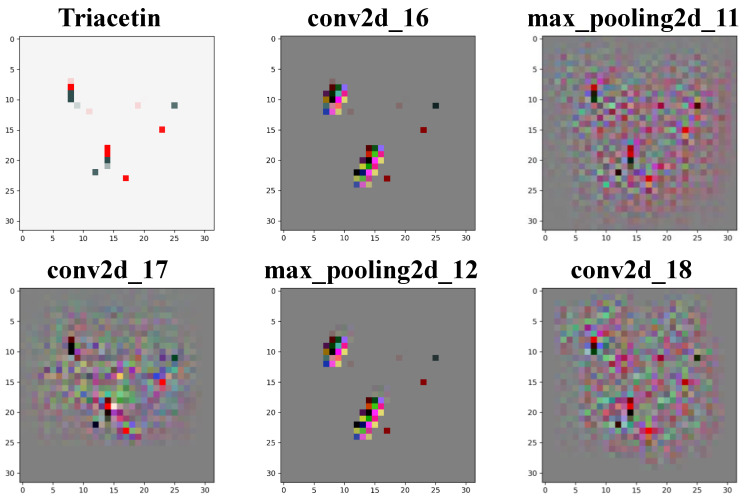
Visualization of the convolution layers (conv2d_16, conv2d_17, and conv2d_18) and the max pooling layers (max_pooling2d_11 and max_pooling2d_12) of Triacetin in the CNN model. The conv2d_16 and max_pooling2d_12 are the detection channel of atomic information of the tested molecule, and the max_pooling2d_11, conv2d_17, and conv2d_18 show the process of extracting key atomic information of the tested molecule.

Taking the visualization of Triacetin as an example (Figure 6), the features are extracted through convolution and pooling. With the increase of the layers, the structural features of Triacetin can be obtained from three dimensions of width, height, and depth (channel). That is, the CNN successfully learned the key features of molecular images, including the molecular skeleton, residues, chemical bonds, etc., and can characterize the correlation between structure features and odor labels. In addition, it is worth emphasizing that, in the process of extracting molecular image characteristics by RDKit [15], hydrogen exists in an implicit form and will not be displayed in the image. As @ or @@ in SMILES denotes that the chiral atom and the surrounding atom were the “clockwise” or “counterclockwise”, respectively [24], thus, the issue of stereoisomerism is also considered in the 2D image of molecules to ensure reasonable characterization of the molecular odor characteristics.

## 5. Conclusions

We constructed three kinds of predictors based on MLP-Des, MLP-Fin, and CNN to predict molecular odors. The results show that the MLP-Des model had the highest accuracy for the binary-classification prediction of odor/no odor, fruity/no odor, floral/no odor, and woody/no odor, as well as the multi-classification prediction of no/fruity/flowery/woody odor. The structure–odor relationship derived from the MLP-Des model revealed that molecular charge, weight, composition, shape, surface interaction, and hydrophobic interaction were closely related to the molecular odor characteristics. The CNN could automatically extract molecular image features, which could avoid large errors caused by screening features according to the designer’s experience; moreover, it was able to establish a close relationship between 2D images and molecular odors.

In the future, with the development of separation and identification technology, more odor datasets will be obtained, which will lay a foundation for establishing more accurate prediction models. How to characterize the molecular structure and improve the modeling method is still an urgent problem that needs to be solved. The application of artificial intelligence provides a powerful tool for the prediction and modeling of various molecular odors. This study gives a theoretical basis for identifying potential molecules with specific odors and a methodological basis for predicting the odors of hazardous components in an environment.

## Figures and Tables

**Table 1:** The odorant datasets used in this study.

Category	Training Set	Test Set	Total	Total *	Source
Odor	1670	417	2087	25,044	FlavorDB [12] PubChem [13]
Fruity	512	128	640	7680	
Floral	255	64	319	3828	
Woody	195	49	244	2928	
Odorless	329	82	411	4932	

* The number of odor molecules after data augmentation processing.

**Table 2:** The prediction performance on the test set by the two-classification models.

Category	Models	Precision	Sensitivity	Specificity	MCC
Odor/Odorless	MLP-Des	0.994	0.996	0.918	0.930
	MLP-Fin	0.971	0.991	0.600	0.684
	CNN	0.985	0.994	0.800	0.836
Fruity/Odorless	MLP-Des	0.993	0.979	0.974	0.931
	MLP-Fin	0.917	0.980	0.640	0.710
	CNN	0.967	0.984	0.857	0.867
Floral/Odorless	MLP-Des	0.987	0.974	0.993	0.962
	MLP-Fin	0.891	0.965	0.765	0.767
	CNN	0.960	0.975	0.917	0.901
Woody/Odorless	MLP-Des	0.970	0.983	0.950	0.938
	MLP-Fin	0.899	0.942	0.835	0.789
	CNN	0.957	0.949	0.931	0.880

**Table 3:** The prediction performance on the test set by the multi-classification (fruity/floral/woody/odorless) models.

Models	Accuracy	Precision	Sensitivity
MLP-Des	0.800	0.802	0.800
MLP-Fin	0.700	0.700	0.701
CNN	0.704	0.710	0.703

**Table 4:** Comparison of the prediction results between our models and other models.

Model	Task	Input	Data Set	Accuracy	*AUC*
AI-RF/DBN [5]	Distinguishing color and odor	Physicochemical features	Test set	0.93	-
				0.94	-
GA-ANN [21]	Evaluating raw beef flavor	Sensor array	Test	0.85	-
	Evaluating cooked beef flavor		Test	0.90	-
MILP/MINLP [4]	Screening fragrance molecules	Group contribution	Train	0.93	-
			Test	0.75	-
DREAM-RF [23]	Predicting the perceived odor of a given molecule	Chemical features	Test	-	0.83
Olfactometer [22]	Predicting odor perception of odorant molecules	Physicochemical features	Calibration	0.97	-
				0.93	-
			Test	0.97	-
SOR [7]	Predicting multi-label of odorant molecules	Fingerprint	Test	0.97	0.78
		Descriptor			
		Image	Test	0.98	0.87
CNN	Classifying odor/odorless molecules	Image	Test set	0.98	0.98
MLP-Des		Descriptor		0.99	0.99
MLP-Fin		Fingerprint		0.98	0.99
CNN	Classifying fruity/odorless molecules	Image	Test set	0.96	0.98
MLP-Des		Descriptor		0.98	0.99
MLP-Fin		Fingerprint		0.91	0.95
CNN	Classifying floral/odorless molecules	Image	Test set	0.96	0.99
MLP-Des		Descriptor		0.98	0.99
MLP-Fin		Fingerprint		0.90	0.93
CNN	Classifying woody/odorless molecules	Image	Test set	0.94	0.98
MLP-Des		Descriptor		0.97	0.99
MLP-Fin		Fingerprint		0.90	0.93
CNN	Classifying fruity/floral/woody/odorless molecules	Image	Test set	0.71	0.80
MLP-Des		Descriptor		0.80	0.86
MLP-Fin		Fingerprint		0.70	0.80

## Data Availability

No new data were created or analyzed in this study. Data sharing is not applicable to this article.

## Supplementary Materials

The following supporting information can be downloaded at: https://www.mdpi.com/article/10.3390/foods11142033/s1, Table S1: Total 200 molecular descriptors calculated by RDKit; Table S2: The optimized parameters used for odor prediction models; Table S3: The prediction performance on the test set by the two-classification models; Table S4: The prediction performance on the test set by the multi-classification (fruity/floral/woody/odorless) models; Table S5: The oblique rotation component matrix in the MLP-Des model for the prediction of odorous/odorless molecules; Table S6: The oblique rotation component matrix in the MLP-Des model for the prediction of odorless/fruity molecules; Table S7: The oblique rotation component matrix in the MLP-Des model for the prediction of odorless/floral molecules; Table S8: The oblique rotation component matrix in the MLP-Des model for the prediction of odorless/woody molecules; Table S9: The oblique rotation component matrix in the MLP-Des model for the prediction of multi-class (fruity/floral/woody/odorless) molecules.
