# Supplementary material for: Insight into the Structure–Odor Relationship of Molecules: A Computational Study Based on Deep Learning
Source: Foods. 2022 Jul 9;11(14):2033. doi: 10.3390/foods11142033 (PMC9320518; doi:10.3390/foods11142033)
Supplement: Supplementary file 1 [file foods-11-02033-s001.zip › foods-1682777-supplementary.pdf]

Table S1. Total 200 molecular descriptors calculated by RDKit (Landrum, 2016)

Descriptor/Descriptor Family	Notes	Language
Gasteiger/Marsili Partial Charges	*Tetrahedron* **36**:3219-28 (1980)	C++
BalabanJ	*Chem. Phys. Lett.* **89**:399-404 (1982)	Python
BertzCT	*J. Am. Chem. Soc.* **103**:3599-601 (1981)	Python
Ipc	*J. Chem. Phys.* **67**:4517-33 (1977)	Python
HallKierAlpha	*Rev. Comput. Chem.* **2**:367-422 (1991)	C++
Kappa1 - Kappa3	*Rev. Comput. Chem.* **2**:367-422 (1991)	C++
Chi0, Chi1	*Rev. Comput. Chem.* **2**:367-422 (1991)	Python
Chi0n - Chi4n	*Rev. Comput. Chem.* **2**:367-422 (1991)	C++
Chi0v - Chi4v	*Rev. Comput. Chem.* **2**:367-422 (1991)	C++
MolLogP	Wildman and Crippen *JCICS* **39**:868-73 (1999)	C++
MolMR	Wildman and Crippen *JCICS* **39**:868-73 (1999)	C++
MolWt		C++
ExactMolWt		C++
HeavyAtomCount		C++
HeavyAtomMolWt		C++
NHOHCount		C++
NOCount		C++
NumHAcceptors		C++
NumHDonors		C++
NumHeteroatoms		C++
NumRotatableBonds		C++
NumValenceElectrons		C++
NumAmideBonds		C++
Num{Aromatic,Saturated,Aliphatic} Rings		C++
Num{Aromatic,Saturated,Aliphatic} {Hetero,Carbo}cycles		C++
RingCount		C++
FractionCSP3		C++
NumSpiroAtoms	Number of spiro atoms(atoms shared between rings that share exactly one atom)	C++
NumBridgeheadAtoms	Number of bridgehead atoms (atoms shared between rings that share at least two bonds)	C++
TPSA	*J. Med. Chem.* **43**:3714-7, (2000) See the section in the RDKit book describing differences to the original publication.	C++
LabuteASA	*J. Mol. Graph. Mod.* **18**:464-77 (2000)	C++
PEOE_VSA1 - PEOE_VSA14	MOE-type descriptors using partial charges and surface area contributions http://www.chemcomp.com/journal/vsadesc.htm	C++
SMR_VSA1 - SMR_VSA10	MOE-type descriptors using MR contributions and surface area contributions http://www.chemcomp.com/journal/vsadesc.htm	C++
SlogP_VSA1 - SlogP_VSA12	MOE-type descriptors using LogP contributions and surface area contributions http://www.chemcomp.com/journal/vsadesc.htm	C++
EState_VSA1 - EState_VSA11	MOE-type descriptors using EState indices and surface area contributions (developed at	Python

	RD, not described in the CCG paper)	
VSA_EState1 - VSA_EState10	MOE-type descriptors using EState indices and surface area contributions (developed at RD, not described in the CCG paper)	Python
MQNs	Nguyen et al. *ChemMedChem* **4**:1803-5 (2009)	C++
Topliss fragments	implemented using a set of SMARTS definitions in $(RDBASE)/Data/FragmentDescriptors.csv	Python
Autocorr2D	New in 2017.09 release. Todeschini and Consoni "Descriptors from Molecular Geometry" Handbook of Chemoinformatics https://doi.org/10.1002/9783527618279.ch37	C++

Table S2. The optimized parameters used for odor prediction models

Category	Models	Learning rate	Dropout	Batch_size
Odor/Odorless	MLP-Des	0.0007	0.1	32
	MLP-Fin	0.0001	0.2	32
	CNN	0.0001	0.1	32
Fruity/Odorless	MLP-Des	0.0007	0.1	32
	MLP-Fin	0.0001	0.2	32
	CNN	0.0001	0.1	32
Floral/Odorless	MLP-Des	0.001	-	20
	MLP-Fin	0.0001	0.2	30
	CNN	0.0001	0.1/0.2	32
Woody/Odorless	MLP-Des	0.001	-	20
	MLP-Fin	0.0001	0.2	32
	CNN	0.0001	0.1/0.2	32
Fruity/Floral/Woody/Odorless	MLP-Des	0.001	0.5	32
	MLP-Fin	0.0001	0.5	32
	CNN	0.0001	0.2	32

Table S3. The prediction performance on the test set by the two-classification models

Category	Models	*Precision*	*Sensitivity*	*Specificity*	*MCC*
	MLP-Des	0.994	0.996	0.918	0.930
Odor/Odorless	MLP-Fin	0.971	0.991	0.600	0.684
	CNN	0.985	0.994	0.800	0.836
Fruity/Odorless	MLP-Des	0.993	0.979	0.974	0.931
	MLP-Fin	0.917	0.980	0.640	0.710
	CNN	0.967	0.984	0.857	0.867
	MLP-Des	0.987	0.974	0.993	0.962
Floral/Odorless	MLP-Fin	0.891	0.965	0.765	0.767
	CNN	0.960	0.975	0.917	0.901
	MLP-Des	0.970	0.983	0.950	0.938
Woody/Odorless	MLP-Fin	0.899	0.942	0.835	0.789
	CNN	0.957	0.949	0.931	0.880

Table S4. The prediction performance on the test set by the multi-classification (fruity/floral/woody/odorless) models

Models	*Accuracy*	*Precision*	*Sensitivity*
MLP-Des	0.800	0.802	0.800
MLP-Fin	0.700	0.700	0.701
CNN	0.704	0.710	0.703

Table S5. The oblique rotation component matrix in the MLP-Des model for the prediction of odorous/odorless molecules

	Components			
	1	2	3	4
MolMR	0.974	-	-	-
VSA_EState9	0.965	-	-	-
NumValenceElectrons	0.942	-	-	-
LabuteASA	0.938	-	-	-
SlogP_VSA2	0.844	-	-	-
SMR_VSA1	0.840	-	0.361	-
SMR_VSA5	0.833	-	-0.464	-
HeavyAtomMolWt	0.804	0.564	-	-
ExactMolWt	0.800	0.562	-	-
MolWt	0.800	0.562	-	-
PEOE_VSA6	0.789	-0.413	-	-
EState_VSA2	0.787	-	-	-
EState_VSA5	0.784	-0.410	-	-
PEOE_VSA7	0.783	-0.416	-	-
SlogP_VSA5	0.780	-	-0.502	-
EState_VSA8	0.773	-0.469	-	-
SlogP_VSA6	0.754	-0.442	-	0.368
SMR_VSA7	0.725	-0.423	-	0.424
TPSA	0.721	0.400	0.407	-
EState_VSA7	0.714	-0.469	-	-
BertzCT	0.622	0.563	-	0.479
Ipc	-	0.548	-	-

Table S6. The oblique rotation component matrix in the MLP-Des model for the prediction of odorless/fruity molecules

	Components						
	1	2	3	4	5	6	7
NumValenceElectrons	0.974	-	-	-	-	-	-
ExactMolWt	0.954	-	-	-	-	-	-
MolWt	0.954	-	-	-	-	-	-
HeavyAtomMolWt	0.942	-	-	-	-	-	-
Chi2v	0.936	-	-	-	-	-	-
Chi3n	0.909	-	-	-	-	-	-
Chi3v	0.905	-	-	-	-	-	-
Chi4n	0.882	-	-	-	-	-	-
Chi4v	0.875	-	-	-	-	-	-
SMR_VSA5	0.772	-	-	-	0.384	-	-
Ipc	0.608	-	-	-	-	-	-
PEOE_VSA8	0.575	-	-	-	-	-	-
EState_VSA9	0.573	0.428	-	-	-	-	-
SMR_VSA6	0.438		-	-	-	-	-
EState_VSA4	0.409	-0.389	-	-	-	-	-
MaxPartialCharge	-	0.881	-	-	-	-	-
MaxAbsPartialCharge	-	0.874	-	-	-	-	-
PEOE_VSA14	-	0.855	-	-	-	-	-
EState_VSA2	-	0.830	-	-	-	-	-
MinEStateIndex	-	-0.823	-	-	-	-	-
MinAbsPartialCharge	-	0.801	-	-	-	-	-
BalabanJ	-	-0.688	-	-	-	-	-
MinPartialCharge	-	-0.676	-	-	-	-	-
SMR_VSA10	-	0.661	-	-	-	-	-
FpDensityMorgan3	-	-0.636	-	0.367	-	-	0.385
HallKierAlpha	-0.475	0.508	-	-	0.424	-	-
PEOE_VSA7	-	-0.488	-	-	-	-	0.364
PEOE_VSA6	-	-0.462	-	-	0.455	-	-0.378
fr_C_O	0.576		0.675	-	-	-	-
fr_C_O_noCOO	0.601	-	0.654	-	-	-	-

fr_ester	0.589	-	0.603	-	-	-	-
PEOE_VSA2	0.552	-	0.581	-	-	-	-
MaxEStateIndex	0.365	-	0.515	0.448	0.370	-0.407	-
MaxAbsEStateIndex	0.365	-	0.515	0.448	0.370	-0.407	-
SlogP_VSA6	-	-	-	0.809	-	-	-
SMR_VSA7	-	-	-	0.805	-0.369	-	-
SlogP_VSA5	0.484	-0.458	-	-	0.626	-	-
FpDensityMorgan1	-0.516	-	0.374	-	-	-	0.551

Table S7. The oblique rotation component matrix in the MLP-Des model for the prediction of odorless/floral molecules

	Components					
	1	2	3	4	5	6
PEOE_VSA14	0.874	-	-	-	-	-
MaxPartialCharge	0.857	-	-	-	-	-
EState_VSA2	0.847	-	-	-	-	-
MaxAbsPartialCharge	0.842	-	-	-	-	-
MolLogP	-0.840	-	-	-	-	-
MinAbsPartialCharge	0.814	-	-	-	-	-
BalabanJ	-0.775	-	-	-	-	-
FpDensityMorgan3	-0.745	-	0.437	-	-	-
SMR_VSA10	0.696	-	-	-	-	-
MinPartialCharge	-0.673	-	-	-	-	0.477
FpDensityMorgan2	-0.667	-	0.552	-	-	-
qed	-0.595	-	-	-	-	-
PEOE_VSA7	-0.560	-	-	0.534	-	-
NumRotatableBonds	-	0.853	-	-	-	-
fr_C_O_noCOO	-	0.771	0.392	-	-	-
fr_C_O	-	0.731	0.468	-	-	-
Kappa2	0.404	0.725	-	-	-	-
PEOE_VSA8	-	0.702	-	-	-	-
PEOE_VSA2	-	0.695	0.458	-	-	-
Ipc	-	0.687	-	-	-	-
SlogP_VSA5	-0.397	0.664	-	0.351	-	-
EState_VSA4	-	0.573	-	-	-	-
HallKierAlpha	0.457	-0.543	-0.386	-	-	-
FpDensityMorgan1	-0.398	-0.425	0.602	-	-	-
EState_VSA8	-0.420	-	-0.422	0.415	0.379	-
PEOE_VSA6	-0.437	0.444	-0.366	-	-0.508	-
SMR_VSA6	-	0.398	-	-0.407	0.458	-0.561

Table S8. The oblique rotation component matrix in the MLP-Des model for the prediction of odorless/woody molecules

	Components					
	1	2	3	4	5	6
MolLogP	0.894	-	-	-	-	-
SMR_VSA1	-0.834	-	-	-	-	-
NumHeteroatoms	-0.831	0.477	-	-	-	-
TPSA	-0.806	0.465	-	-	-	-
MinEStateIndex	0.803	-	-	-	-	-
FpDensityMorgan3	0.795	-	-	-	0.376	-
MinAbsPartialCharge	-0.782	-	-	-	-	-
PEOE_VSA14	-0.781	-	0.419	-	-	-
MaxPartialCharge	-0.764	-	0.426	-	-	-
MaxAbsPartialCharge	-0.763	-	0.394	-	-	-
FpDensityMorgan2	0.751	-	-	-	0.469	-
EState_VSA2	-0.743	-	0.460	-	-	-
PEOE_VSA7	0.702	-	-	-	-	-
MinPartialCharge	0.678	-	-	-	-	-0.444
QED	0.670	-	-	-	-	-
SMR_VSA10	-0.649	-	0.378	-	-	-
BalabanJ	0.626	-	-0.404	-	-	-
EState_VSA8	0.601	-	-	-	-	-
SlogP_VSA4	0.542	-	0.401	-0.503	-	-
PEOE_VSA6	0.531	0.483	0.387	-	-	-
FpDensityMorgan1	0.521	-0.459	-	-	0.512	-
SMR_VSA4	0.521	-	0.379	-0.483	-	-
NumRotatableBonds	-	0.699	-	0.474	-	-
Kappa2	-0.590	0.642	-	-	-	-
SlogP_VSA5	0.519	0.628	0.444	-	-	-
EState_VSA10	-0.592	0.616	-	-	-	-
RingCount	-	0.601	-	-0.515	-	-
EState_VSA4	-	0.566	-	-	-	-
Ipc	-	0.523	-	0.475	0.366	-
FractionCSP3	0.390	0.513	-	-	-	-

SMR_VSA6	-0.382	0.513	-	-	-	0.353
EState_VSA5	0.443	0.496	0.393	-	-	-
NumHDonors	-0.474	0.446	-0.562	-	-	-
PEOE_VSA2	-	0.431	-	0.578	0.380	-
PEOE_VSA9	-0.438	0.370	-0.367	-	-	0.526

Table S9. The oblique rotation component matrix in the MLP-Des model for the prediction of multi-class (fruity/floral/woody/odorless) molecules

	Components									
	1	2	3	4	5	6	7	8	9	10
MolLogP	-0.864	-	-	-	-	-	-	-	-	-
SMR_VSA1	0.853	-	-	-	-	-	-	-	-	-
TPSA	0.820	0.365	-	-	-	-	-	-	-	-
PEOE_VSA14	0.806	-	-	-	-	-	-	-	-	-
MaxPartialCharge	0.794	-	-	-	0.351	-	-	-	-	-
MinEStateIndex	-0.793	-	-	-	-	-	-	-	-	-
EState_VSA2	0.789	-	-	-	-	-	-	-	-	-
MaxAbsPartialCharge	0.780	-	-	-	-	-	-	-	-	-
MinAbsPartialCharge	0.757	-	-	-	0.360	-	-	-	-	-
SlogP_VSA2	0.678	0.501	-	-	-	-	-	-	-	-
					0.403					
SMR_VSA10	0.664	-	-	-	-	-	-	-	-	-
FpDensityMorgan3	-0.661	-	0.368	-	-	-	-	-	-	-
BalabanJ	-0.661	-	-	-	-	-	-	-	-	-
EState_VSA10	0.610	0.567	-	-	-	-	-	-	-	-
FpDensityMorgan2	-0.609	-	0.385	-	-	0.457	-	-	-	-
MinPartialCharge	-0.604	-	-	-	-	-	-	0.408	-	-
PEOE_VSA7	-0.502	0.448	-	-	-	-	-	-	-	-
PEOE_VSA6	-0.446	0.431	-	-	0.385	-	-	-	-	-
						0.363				
MolMR	-	0.971	-	-	-	-	-	-	-	-
Chi0	-	0.927	-	-	-	-	-	-	-	-
Chi3v	-	0.862	-	-	-	-	-	-	-	-
Chi4n	-	0.851	-	-	-	-	-	-	-	-
SMR_VSA5	-	0.813	-	-	-	-	-	-	-	-
				0.428						
BertzCT	-	0.772	-	0.462	-	-	-	-	-	-
Chi4v	-	0.745	-	-	-	-	-	-	-	-
			0.365							
fr_ether	-	0.632	-	-	-	-	-	-	-	-
					0.439					
NumRotatableBonds	-	0.614	-	-	-	-	-	-	-	-
						0.359				
SlogP_VSA5	-0.423	0.590	-	-	0.532	-	-	-	-	-

HallKierAlpha	-	-0.524	-0.489	-0.392	-	-	-	-	-	-
EState_VSA4	-	0.500	-	-	-	-	-	-	-	-
MaxAbsEStateIndex	-	-	0.676	-	-	-	-	-	-	-
MaxEStateIndex	-	-	0.676	-	-	-	-	-	-	-
fr_C_O	0.366	0.366	0.580	-	-	-	-	0.486	-	-
fr_ester	-	0.443	0.566	-	-	-	-		-0.368	-
fr_C_O_noCOO	-	0.421	0.566	-	-	-	-	0.508	-	-
EState_VSA8	-0.412	0.391	-0.454	-	-	-	-	-	-	-
SMR_VSA7	-	-	-	0.885	-	-	-	-	-	-
SlogP_VSA6	-	-	-	0.879	-	-	-	-	-	-
FractionCSP3	-	-	-	-0.752	-	-	-	-	-	-
SMR_VSA6	-	0.398	-	-	-0.431	-	-	-	-	0.391
FpDensityMorgan1	-0.406	-0.406	-	-	-	0.530	-	-	-	-
SMR_VSA4	-	-	-0.368	-	-	0.473	-	-	-	-
Kappa2	-	-	-	-	-	-0.490	0.781	-	-	-
Kappa1	-	-	-	-	-	-0.455	0.779	-	-	-
fr_allylic_oxid	-0.394	-	-	-	-	-	-	0.362	0.417	-

References

Landrum, G. (2016). Rdkit: Open-source cheminformatics software. *GitHub and SourceForge, 10*, 3592822.
